# HMGCR gene polymorphism is associated with residual cholesterol risk in premature triple-vessel disease patients treated with moderate-intensity statins

**DOI:** 10.1186/s12872-023-03285-w

**Published:** 2023-06-24

**Authors:** Jiawen Li, Xiaofang Tang, Jingjing Xu, Ru Liu, Lin Jiang, Lianjun Xu, Jian Tian, Xinxing Feng, Yajie Wu, Yin Zhang, Dong Wang, Kai Sun, Bo Xu, Wei Zhao, Rutai Hui, Runlin Gao, Lei Song, Jinqing Yuan, Xueyan Zhao

**Affiliations:** National Clinical Research Center for Cardiovascular Diseases, State Key Laboratory of Cardiovascular Disease, Fu Wai Hospital, National Center for Cardiovascular Diseases, Chinese Academy of Medical Sciences and Peking Union Medical College, No. 167 Beilishi Road, Xicheng District, Beijing, 100037 China

**Keywords:** Gene polymorphisms, Residual cholesterol risk, Low-density lipoprotein cholesterol, Premature triple-vessel disease

## Abstract

**Background:**

To investigate the association of *HMGCR* and *NPC1L1* gene polymorphisms with residual cholesterol risk (RCR) in patients with premature triple-vessel disease (PTVD).

**Methods:**

Three SNPs within *HMGCR* including rs12916, rs2303151, and rs4629571, and four SNPs within *NPC1L1* including rs11763759, rs4720470, rs2072183, and rs2073547 were genotyped. RCR was defined as achieved low-density lipoprotein cholesterol (LDL-C) concentrations after statins higher than 1.8 mmol/L (70 mg/dL).

**Results:**

Finally, a total of 609 PTVD patients treated with moderate-intensity statins were included who were divided into two groups: non-RCR group (n = 88) and RCR group (n = 521) according to LDL-C concentrations. Multivariate logistic regression showed the homozygotes for the minor allele of rs12916 within *HMGCR* gene (*CC*) were associated with a 2.08 times higher risk of RCR in recessive model [odds ratio (OR): 2.08, 95% confidence interval (CI): 1.16–3.75]. In codominant model, the individuals homozygous for the minor allele of rs12916 (*CC*) were associated with a 2.26 times higher risk of RCR (OR: 2.26, 95% CI: 1.16–4.43) while the heterozygous individuals (*CT*) were not, compared with the individuals homozygous for the major allele of rs12916 (*TT*). There was no significant association between the SNPs within *NPC1L1* gene and RCR in various models.

**Conclusions:**

We first reported that the variant homozygous *CC* of rs12916 within *HMGCR* gene may incur a significantly higher risk of RCR in PTVD patients treated with statins, providing new insights into early individualized guidance of precise lipid-lowering treatment.

**Supplementary Information:**

The online version contains supplementary material available at 10.1186/s12872-023-03285-w.

## Introduction

Studies on atherosclerotic cardiovascular diseases (ASCVD) in China (2021) reported that the burden of ASCVD in the Chinese population is heavy, and the prevalence of ASCVD is keeping rising. It is estimated that the prevalence of ASCVD is 330 million, including 11.39 million coronary heart disease (CHD). Cardiovascular death accounted for the first place in the all-cause death among urban and rural residents in China [1]. Statins can significantly reduce low-density lipoprotein cholesterol (LDL-C) concentrations and adverse events of ASCVD, which is considered the cornerstone of ASCVD therapy [2]. However, there are individual variations in the efficacy of statins. The LDL-C concentrations after statins in many ASCVD patients cannot accomplish the target recommended by the guideline on the management of blood cholesterol [3], which is related to poor prognosis. Numerous large-scale studies have demonstrated [4–6] that the addition of non-statin lipid-lowering drugs such as ezetimibe and proprotein convertase subtilisin-kexin type 9 inhibitor (PCSK9i) in patients with LDL-C concentrations higher than 1.8 mmol/L (70 mg/dL) on basis of statins can further decrease the risk of cardiovascular events. Consequently, experts put forward the concept of residual cholesterol risk (RCR), which is defined as the achieved LDL-C concentrations higher than 1.8 mmol/L (70 mg/dL) after statins [7, 8]. Exactly as there are individual variations of the efficacy after clopidogrel, and the CYP2C19 genetic testing has been extensively used clinically to early identify patients with high platelet reactivity. Similarly, we wonder whether specific genetic testing can also early identify patients with RCR (LDL-C > 1.8 mmol/L) after statins and this is a topic worthy of investigation. Yet there is a paucity of relevant reports internationally, making early individualized guidance of precise intensive or combined lipid-lowering treatment become a clinical problem.

3-hydroxy-3-methylglutaryl-coenzyme A reductase (HMGCR) and Niemann-Pick C1-like 1 (NPC1L1) are both crucial genes related to lipid metabolism. *HMGCR* gene is involved in endogenous cholesterol synthesis and *NPC1L1* gene is involved in exogenous cholesterol absorption. Previous studies established that *HMGCR* and *NPC1L1* gene polymorphisms were associated with baseline LDL-C concentrations [9], and HMGCR inhibitors (statins) and NPC1L1 inhibitors (ezetimibe) can reduce LDL-C concentrations and the risk of cardiovascular disease [4, 10]. Our previous study [11] showed that *HMGCR* and *NPC1L1* gene polymorphisms were associated with increased adverse events risk in patients with coronary triple-vessel disease (TVD). Nevertheless, there have been few studies that link *HMGCR* and *NPC1L1* gene polymorphisms to RCR (LDL-C > 1.8 mmol/L). Patients with premature triple-vessel disease (PTVD) displayed a higher risk of major adverse cardiovascular events [12] and were usually related to genetic risk factors. The present study aimed to investigate the association of *HMGCR* (the target gene of statins) and *NPC1L1* (the target gene of ezetimibe) with RCR (LDL-C > 1.8 mmol/L), exploring the single nucleotide polymorphisms (SNPs) that can identify patients with a high risk of RCR (LDL-C > 1.8 mmol/L), which will be of great significance to individualized lipid-lowering therapy.

## Methods

### Study populations

A total of 8943 patients diagnosed with TVD by coronary angiography were consecutively enrolled between 2004 and 2011 in Fu Wai Hospital (Beijing, China), of whom 1792 patients (20.0%) were PTVD. Among them, 872 had blood samples and met the criteria of DNA testing.

609 patients treated with statins before at least 1 week were finally enrolled in the study. According to LDL-C concentrations, the 609 subjects were divided into two groups: non-RCR group (LDL-C ≤ 1.8 mmol/L after statins) and RCR group (LDL-C > 1.8 mmol/L after statins). All of them were prescribed moderate-intensity statins but no ezetimibe or PCSK9i previously.

The study complied with the principles of the Declaration of Helsinki and was approved by the Review Board of Fu Wai Hospital. Written informed consent was obtained from all participants.

### Definitions

TVD was defined as angiographically confirmed stenosis of ≥ 50% in all three main epicardial coronary arteries (left anterior descending, left circumflex, and right coronary arteries), with or without the involvement of the left main artery. PTVD was defined as male patients with TVD ≤ 50 years old and female patients with TVD ≤ 60 years old [13]. RCR was defined as the achieved LDL-C concentrations after statins higher than 1.8 mmol/L (70 mg/dL) [7, 8]. The definition of moderate-intensity statins was a regime of daily doses that can reduce LDL-C by about 30–50% including atorvastatin 10–20 mg/day, rosuvastatin 5–10 mg/day, simvastatin 20–40 mg/day, pivastatin 2–4 mg/day or fluvastatin 80 mg/day [14].

### Selection of SNPs and genotyping assays

*NPC1L1* is located in chromosome 7, and *HMGCR* is located in chromosome 5. In the HapMap database (http://www.hapmap.org) for Chinese Han Beijing (CHB) adults using the algorithm-Tagger-pairwise tagging method, three tag SNPs in *NPC1L1* (rs11763759, rs4720470, and rs2072183) were picked out for population CHB chr7: 44,518,661–44,547,439; and three tag SNPs in *HMGCR* (rs12916, rs2303152, and rs2303151) were picked out for population CHB chr5: 74,668,855–74,693,680. Minor allele frequency and the determinant coefficient (r^2^) thresholds were set at 0.05 and 0.8, respectively. After consulting the previous works of literature, we added one SNP (rs2073547) in *NPC1L1* and one SNP (rs4629571) in *HMGCR* [9]. After patient specimens were tested, we removed rs2303152 due to too much missing data in our subjects. Finally, 7 SNPs were determined: four SNPs in *NPCIL1* gene (rs11763759, rs4720470, rs2072183, and rs2073547) and three SNPs in *HMGCR* gene (rs12916, rs2303151, and rs4629571).

Fasting blood samples from all subjects were taken within 24 h after admission to establish a blood dataset to extract DNA. Genomic DNA was extracted from leukocytes through the standard salting-out method [15]. In this study, the SNP genotyping work was performed using an improved multiplex ligation detection reaction (iMLDR) technique which was newly developed by Genesky Biotechnologies Inc. (Shanghai, China) [16] with a custom-by-design 48-Plex SNPscan™ Kit (Cat#:G0104; Genesky Biotechnologies Inc., Shanghai, China). This kit was developed according to patented SNP genotyping technology by Genesky Biotechnologies Inc., which was based on double ligation and multiplex fluorescence PCR. All probes were designed by and ordered from Genesky Biotechnologies Inc. (Shanghai, China). Our actual steps were illustrated in Supplementary method.

### Laboratory index measurement

Plasma glucose was measured using the glucose assay kit (Biosino Bio-Technology And Science Incorporation, Beijing, China) with glucose oxidase method. Total cholesterol (TC) with CHOD-PAP method, and triglycerides (TGs) with GPO-PAP method were determined using corresponding commercially available test kits (Biosino Bio-Technology And Science Incorporation, Beijing, China). Plasma high-density lipoprotein cholesterol (HDL-C) with chemistry modify enzyme method and LDL-C with selective melt method were determined using corresponding commercially available test kits (Minaris Medical (Shanghai) Co., Ltd., Shanghai, China). Serum creatinine was determined using creatinine assay kit with sarcosine oxidase method (Weihai Weigao Biotech Co., Ltd., Shandong, China). Analyses were conducted on an automatic biochemical analyzer (Hitachi 7150, Hitachi Group, Japan). All other laboratory measurements were conducted at the biochemistry center of Fu Wai Hospital by standard biochemical techniques.

### Statistical analysis

Continuous variables were expressed as mean ± standard deviation. Student’s t-test was used to compare continuous variables between two groups. Categorical variables were expressed as numbers (%). We tested conformity of the *NPC1L1* and *HMGCR* gene polymorphisms to Hardy–Weinberg equilibrium (HWE) among the enrolled patients using the chi-square test or Fisher’s exact test. The two-sided *P* value > 0.05 was considered in conformity with HWE. Pearson χ^2^ test or Fisher’s exact test was used to compare categorical variables between two groups. Comparisons of frequency of genotypes between non-RCR and RCR groups in three genetic models including dominant, recessive and, codominant models were conducted by Pearson χ^2^ test or Fisher’s exact test. Subsequently, the Bonferroni test was used for a multiple comparison procedure in codominant model. Univariable and multivariable logistic regressions were performed to evaluate the association between genotypes of SNP and RCR, and the results were reported as odds ratio (OR) and 95% confidence interval (CI). The multivariable model was adjusted for age and sex. Statistical significance was defined as two-sided *P* values of < 0.05. All analyses were performed using SPSS software version 23.0 (IBM, Armonk, NY, USA).

## Results

### Baseline characteristics

**Fig. 1:**
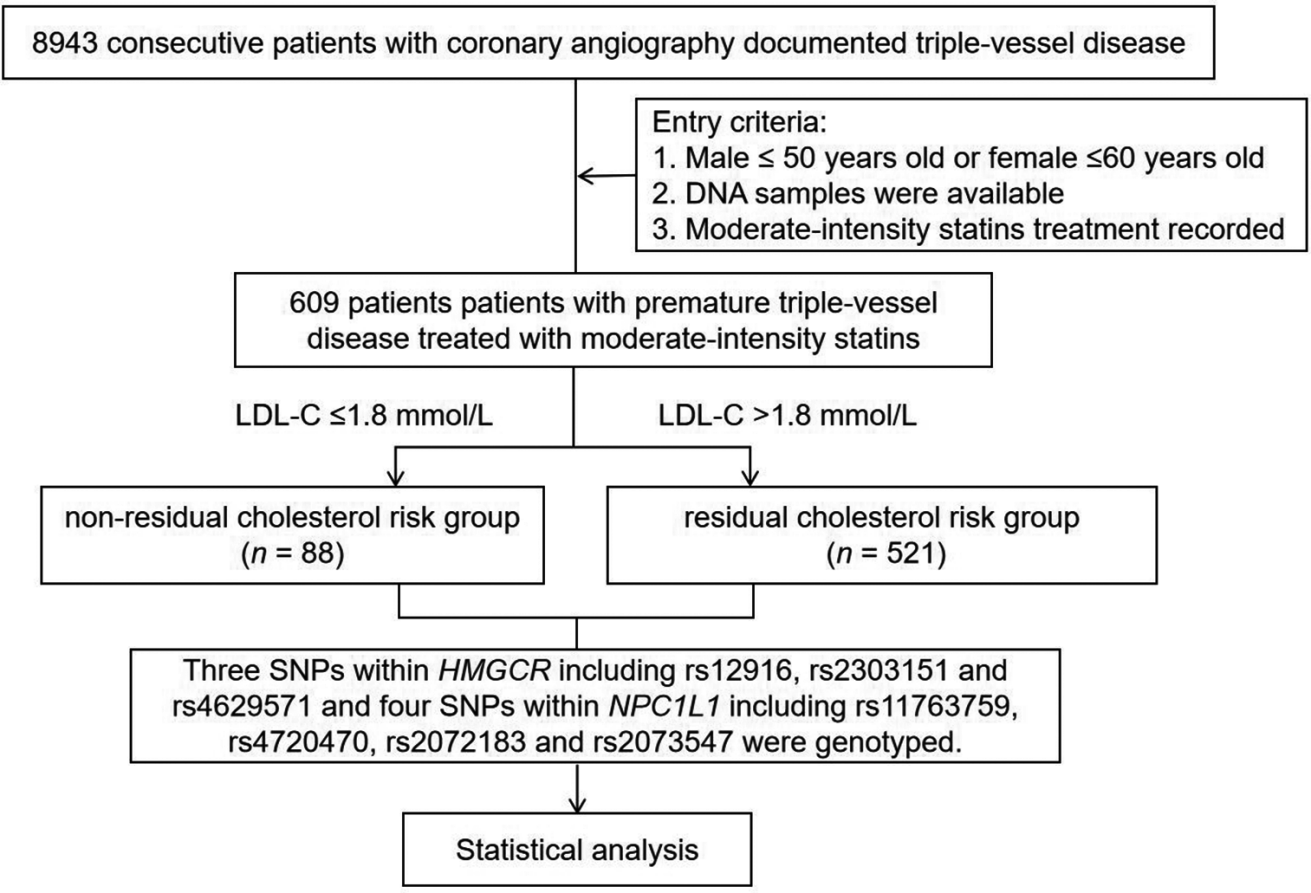
Patient Flow Chart DNA, deoxyribonucleic acid; LDL-C, low-density lipoprotein cholesterol; SNPs, single nucleotide polymorphisms; HMGCR, 3-hydroxy-3-methylglutaryl-coenzyme A reductase; NPC1L1, Niemann-Pick C1-like 1

A total of 609 PTVD patients treated with moderate-intensity statins were included in the analysis (Fig. 1). The mean age was 47.3 ± 6.2 years and 457 (75.3%) were male patients. Table 1 shows the comparison of baseline characteristics of patients with RCR (LDL-C > 1.8 mmol/L) (n = 521, 85.5%) versus patients without RCR (LDL-C ≤ 1.8 mmol/L) (n = 88, 14.5%). Patients in RCR group tended to have higher total cholesterol, LDL-C and non-high-density lipoprotein cholesterol (*P* < 0.05). There was no significant difference in other baseline data (*P* > 0.05).

**Table 1:** Baseline Characteristic

Parameters	Non-RCR group (*n* = 88)	RCR group (*n* = 521)	*P* value
Male, %	70 (79.5)	387 (74.3)	0.291
Age, %	46.4 ± 6.4	47.4 ± 6.1	0.156
Diabetes mellitus, %	34 (38.6)	186 (35.7)	0.596
Smoking history, %	48 (54.5)	296 (56.8)	0.691
BMI, kg/m^2^	26.7 ± 3.3	26.4 ± 3.0	0.438
Systolic pressure, mmHg	126.8 ± 16.7	123.6 ± 18.3	0.124
Diastolic pressure, mmHg	80.2 ± 12.0	78.2 ± 11.7	0.155
Heart rate, /min	68.8 ± 9.9	71.0 ± 10.3	0.062
Glucose, mmol/L	6.1 ± 1.8	6.2 ± 2.1	0.494
Serum creatinine, mmol/L	79.7 ± 15.5	78.0 ± 16.4	0.360
TG, mmol/L	2.3 ± 1.7	2.0 ± 1.0	0.151
TC, mmol/L	3.6 ± 0.7	5.0 ± 1.0	< 0.001
HDL-C, mmol/L	1.0 ± 0.2	1.0 ± 0.2	0.062
LDL-C, mmol/L	1.6 ± 0.2	2.9 ± 0.8	< 0.001
non-HDL-C, mmol/L	2.6 ± 0.7	4.0 ± 1.0	< 0.001

***Note.*** *RCR, residual cholesterol risk; BMI, body mass index; TG, Triglyceride; TC, total cholesterol; HDL-C, high-density lipoprotein cholesterol; LDL-C, low-density lipoprotein cholesterol; non-HDL-C, non-high-density lipoprotein cholesterol*

### Association analysis of *HMGCR* gene polymorphisms

All SNPs in *HMGCR* gene in overall enrolled patients conformed to Hardy-Weinberg equilibrium (*P* > 0.05) (Table S1). During 3 SNPs (rs12916, rs2303151, and rs4629571), only the frequency of genotype of rs12916 was significantly different between RCR and non-RCR groups in recessive model (RCR_*CC*/*CT+TT*_: 30.1%/69.9% vs. non-RCR_*CC*/*CT+TT*_: 17.0%/83.0%, *P* = 0.012) and codominant model (RCR_*CC*/*CT*/*TT*_: 30.1%/45.1%/24.8% vs. non-RCR_*CC*/*CT*/*TT*_: 17.0%/51.1%/31.8%, *P* = 0.037) (Table 2). At the same time, the multiple comparisons in codominant model showed that the significant difference attributes to the different rates of RCR (*CC* vs. *TT*) between individuals homozygous for the minor allele of rs12916 (*CC*) and individuals homozygous for the major allele of rs12916 (*TT*). (not shown in the Table).

**Table 2:** Frequency of Gene Polymorphisms

Gene	SNPs	Position	Function	Genetic model	Non-RCR group (*n* = 88)	RCR group (*n* = 521)	*P* value
*NPC1L1*	rs11763759	44570067	Intron10	DO: *TT* / *CC* + *CT*	78(88.6%) / 10(11.4%)	471(90.4%) / 50(9.6%)	0.607
				RE: *CC* / *CT* + *TT*	1(1.1%) / 87(98.9%)	2(0.4%) / 519(99.6%)	0.374
				CO: *CC* / *CT* / *TT*	1(1.1%) / 9(10.2%) / 78(88.6%)	2(0.4%) / 48(9.2%) / 471(90.4%)	0.379
	rs4720470	44561884	Intron10	DO: *CC* / *TT* + *CT*	47(53.4%) / 41(46.6%)	239(45.9%) / 282(54.1%)	0.190
				RE: *TT* / *CT* + *CC*	12(13.6%) / 76(86.4%)	57(10.9%) / 464(89.1%)	0.461
				CO: *TT* / *CT* / *CC*	12(13.6%) / 29(33.0%) / 47(53.4%)	57(10.9%) / 225(43.2%) / 239(45.9%)	0.194
	rs2072183	44579180	Exon2	DO: *GG* / *CC* + *GC*	35(39.8%) / 53(60.2%)	207(39.7%) / 314(60.3%)	0.994
				RE: *CC* / *GC* + *GG*	13(14.8%) / 75(85.2%)	65(12.5%) / 456(87.5%)	0.551
				CO: *CC* / *GC* / *GG*	13(14.8%) / 40(45.5%) / 35(39.8%)	65(12.5%) / 249(47.8%) / 207(39.7%)	0.820
	rs2073547	44582331	5’ Flanking	DO: *AA* / *GG* + *GA*	34(38.6%) / 54(61.4%)	207(39.7%) / 314(60.3%)	0.846
				RE: *GG* / *GA* + *AA*	13(14.8%) / 75(85.2%)	66(12.7%) / 455(87.3%)	0.587
				CO: *GG* / *GA* / *AA*	13(14.8%) / 41(46.6%) / 34(38.6%)	66(12.7%) / 248(47.6%) / 207(39.7%)	0.862
*HMGCR*	rs12916	74656539	3’ UTR	DO: *TT* / *CC* + *CT*	28(31.8%) / 60 (68.2%)	129(24.8%) / 392(75.2%)	0.162
				RE: *CC* / *CT* + *TT*	15(17.0%) / 73(83.0%)	157(30.1%) / 364(69.9%)	0.012
				CO: *CC* / *CT* / *TT*	15(17.0%) / 45(51.1%) / 28(31.8%)	157(30.1%) / 235(45.1%) / 129(24.8%)	0.037
	rs2303151	74655451	Intron5	DO: *CC* / *TT* + *CT*	61(69.3%) / 27(30.7%)	335(64.3%) / 186(35.7%)	0.361
				RE: *TT* / *CT* + *CC*	1(1.1%) / 87(98.9%)	20(3.8%) / 501(96.2%)	0.340
				CO: *TT* / *CT* / *CC*	1(1.1%) / 26(29.5%) / 61(69.3%)	20(3.8%) / 166(31.9%) / 335(64.3%)	0.460
	rs4629571	74658304	3’ Flanking	DO: *AA* / *GG* + *GA*	72(81.8%) / 16(18.2%)	428(82.1%) / 93(17.9%)	0.940
				RE: *GG* / *GA* + *AA*	2(2.3%) / 86(97.7%)	4(0.80%) / 517(99.2%)	0.210
				CO: *GG* / *GA* / *AA*	2(2.3%) / 14(15.9%) / 72(81.8%)	4(0.80%) / 89(17.1%) / 428(82.1%)	0.336

***Note.*** *RCR, residual cholesterol risk; HMGCR, 3-hydroxy-3-methylglutaryl-coenzyme A reductase; NPC1L1, Niemann-Pick C1-like 1; DO, dominant model; RE, recessive model; CO, codominant model*

**Fig. 2:**
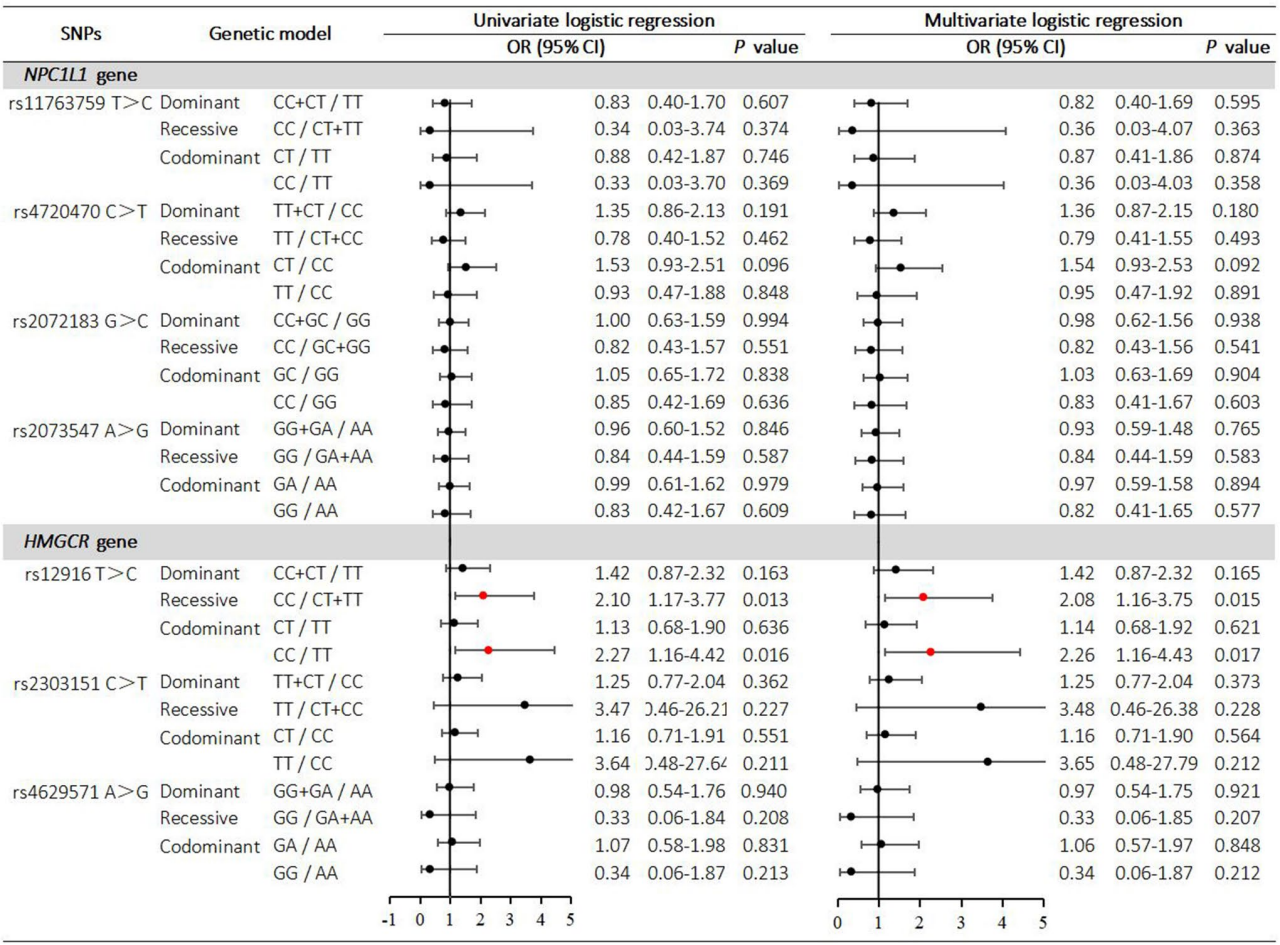
Logistic regression analysis between HMGCR and NPCL1L gene polymorphisms and residual cholesterol risk HMGCR, 3-hydroxy-3-methylglutaryl-coenzyme A reductase; NPC1L1, Niemann-Pick C1-like 1; OR, odds ratio; CI, confidential interval

In recessive model, *C* variant homozygous patients of rs12916 (*CC* vs. *CT* + *TT*)[adjusted OR (OR_adj_): 2.08, 95% CI: 1.16–3.75; *P* = 0.015] were associated with higher risk of RCR (LDL-C > 1.8 mmol/L), including after multivariable logistic regression adjusted for age and sex (Fig. 2). In codominant model, *C* variant homozygous patients of rs12916 (*CC* vs. *TT*) (OR_adj_: 2.26, 95% CI: 1.16–4.43; *P* = 0.017) were associated with higher risk of RCR (LDL-C > 1.8 mmol/L) but *C* variant heterozygous patients of rs12916 (*CT* vs. *TT*) (OR_adj_: 1.14, 95% CI: 0.68–1.92; *P* = 0.621) were not associated with higher risk of RCR (LDL-C > 1.8 mmol/L), including after multivariable logistic regression adjusted for age and sex (Fig. 2). In dominant model, *C* variant homozygous and heterozygous patients of rs12916 (*CC* + *CT* vs. *TT*) (OR_adj_: 1.42, 95% CI: 0.87–2.32; *P* = 0.165) were not associated with higher risk of RCR (LDL-C > 1.8 mmol/L), including after multivariable logistic regression adjusted for age and sex (Fig. 2).

Univariate and multivariable logistic regressions showed other SNPs (rs2303151 and rs4629571) within *HMGCR* were not significantly associated with RCR (LDL-C > 1.8 mmol/L) in three models.

### Association analysis of *NPC1L1* gene polymorphisms

All SNPs in *NPC1L1* gene in overall enrolled patients conformed to Hardy-Weinberg equilibrium (*P* > 0.05) (Table S1). There was no significant difference in the frequency of genotype of 4 SNPs including rs11763759, rs4720470, rs2072183, and rs2073547 (*P* > 0.05) (Table 2).

Univariate and multivariable logistic regressions showed that there was no significant association between the 4 SNPs within *NPC1L1* and RCR (LDL-C > 1.8 mmol/L) in PTVD patients treated with long-term moderate-intensity statins in three models (*P* > 0.05) (Fig. 2).

### Sex-specific difference

We further carried out the interaction of sex*gene polymorphisms (*NPC1L1* and *HMGCR*) on the risk of RCR. There was no sex - gene polymorphisms interaction under any models in the overall population (all p for interaction > 0.05), indicating that the result was consistent in women and men.

## Discussion

There are individual variations in the efficacy of statins in many ASCVD patients. We tested SNPs in two genes (*HMGCR* and *NPC1L1*) related to dyslipidemia in PTVD patients treated with moderate-intensity statins, and investigated the association between their SNPs and RCR (LDL-C > 1.8 mmol/L). The results showed that (1) We proved for the first time that the homozygote (*CC*) for the minor allele of rs12916 within *HMGCR* gene was the independent risk factor of RCR (LDL-C > 1.8 mmol/L) in PTVD patients treated with moderate-intensity statins, and the risk of RCR (LDL-C > 1.8 mmol/L) in genotype *CC* carriers increased to 2.08 times in recessive model and 2.26 times in codominant model; (2) No statistically significant association was observed between other SNPs (rs2303151 and rs4629571) within *HMGCR* gene and four SNPs (rs11763759, rs4720470, rs2072183, and rs2073547) within *NPC1L1* gene and RCR (LDL-C > 1.8 mmol/L) in PTVD patient treated with moderate-intensity statins (without receiving ezetimibe or PCSK9i) in three models.

*HMGCR* gene located in chromosome 5 encodes HMGCR proteins, a rate-limiting enzyme of endogenous cholesterol synthesis and the target of statins. By inhibiting HMGCR protein, statins can reduce cholesterol synthesis in the liver, upregulate LDL-C receptors and lower cardiovascular risk [2]. *NPC1L1* gene located in chromosome 7 encodes NPC1L1 proteins, a key protein for cholesterol absorption in intestine and the target of ezetimibe. By inhibiting NPC1L1 protein, ezetimibe can reduce exogenous cholesterol absorption and then lower the cardiovascular risk [4]. It’s reported that both *HMGCR* and *NPC1L1* genes had many functional effects [17]. Since PTVD poses a significant threat to human health in the world, early diagnosis and timely and aggressive therapeutic intervention are particularly important. Investigating the genetic variation of CHD has important guiding significance for exploring its mechanism, screening high-risk patients with premature CHD, and improving treatment strategies. Our previous study showed that *HMGCR* and *NPC1L1* gene polymorphisms were associated with the susceptibility of PTVD [18] and the risk of diabetes in PTVD [19]. However, the data about the association of the two genes with PTVD was still scarce. Previous studies demonstrated that alternative splicing of *HMGCR* was associated with individual variability in statin efficacy, [20, 21] and the variant *HMGCR* gene was not only associated with LDL-C concentrations [9] but also raised CHD risk, particularly in patients with severe coronary artery disease [11]. Given there is a lack of association analysis of *HMGCR* and *NPC1L1* gene polymorphisms with RCR (LDL-C > 1.8 mmol/L), the present study aimed to examine the association of *HMGCR* and *NPC1L1* gene polymorphisms with RCR (LDL-C > 1.8 mmol/L) in patients with PTVD and found that variant *HMGCR* gene can cause an increased risk of RCR, which provides knowledge of new genetic factors that predispose to cardiovascular diseases. The clinical value of our study lies in the early genetic detection to identify those who may be less likely to achieve LDL-C < 1.8 mmol/L after statins, providing clues for the precise treatment of drugs in PTVD patients. The differences in the observed and expected values of the distributed frequencies of the three genotypes were not significant, which showed that the sample is from a large group in random mating equilibrium and is representative.

Several studies reported the association analysis between *HMGCR* gene polymorphisms and reduction in LDL-C response to statins. Chasman et al. [22] found that SNP12 and SNP29 within *HMGCR* gene were associated with the lower efficacy of pravastatin. Krauss et al. [23] found that H7 and/or H2 (haplotypes within *HMGCR*) carriers were associated with the lower efficacy of simvastatin. However, there were also some studies with opposite results. Polisecki et al. [24] tested *HMGCR* intron 18 *T* > *G* (rs17238540) and found that there was no association between these rare allele carriers and the efficacy of pravastatin. Donnelly et al. [25] found that there was no association between rs17238540 within *HMGCR* and the efficacy of various statins (atorvastatin, cerivastatin, fluvastatin, rosuvastatin, and simvastatin). Besides, only the study conducted by Singer et al. [26] contained an SNP in our study (rs12916) and found that there was no association between five SNPs (rs17244841, rs5908, rs17238540, rs12916, and Dletion) and the efficacy of fluvastatin in renal transplant recipients, which was inconsistent with our results. The following reasons may explain the discrepancy: (1) Different study populations. Singer et al. [26]. enrolled renal transplant recipients while we enrolled patients with PTVD [rs12916-(*T* vs. *C*): 594:624; and rs12916-(*TT* vs. *TC* vs. *CC*): 157:280:172]; (2) Different evaluation endpoints. We took LDL-C concentrations below 1.8 mmol/L as the evaluation endpoint rather than the magnitude of the LDL-C reduction response to statins, because the former is the target of statins and is a more accurate indicator of the efficacy of statins; (3) Different ethnic groups. The different genes between Asian and European and American populations may have effects on the results. It’s worth mentioning that the magnitude of LDL-C reduction after the use of statins was regarded as the evaluation endpoint in all of the studies above-mentioned, however, the current guidelines place more emphasis on the target level of LDL-C (< 1.8 mmol/L) after the use of lipid-lowering drugs than the magnitude of LDL-C reduction.

The SNP rs12916 in *HMGCR* is located on human chr 5: 74,656,539, belonging to the 3 prime UTR variant. It has been repeatedly established that rs12916 was associated with LDL-C in genome-wide association analysis and candidate gene association studies. Previous studies [27, 28] confirmed that the rs12916-*T* allele in *HMGCR* was closely associated with lower expression of HMGCR proteins and lower circulating LDL-C concentrations in the liver. In other words, the rs12916-*C* allele in *HMGCR* was associated with higher HMGCR proteins and higher circulating LDL-C concentrations in the liver. Moreover, rs12916 in the HMGCR gene was associated with the coagulation function [29], higher risk of type 2 diabetes mellitus [19, 30], and serum LDL-C [31, 32] and other concentrations of apoprotein B-containing lipoproteins levels [32]. Rs12916-*T*, which was associated with lower HMGCR expression mimics the well-known effects of statins and is used as the instrumental variable to proxy statin treatment in the Mendelian randomization study [32]. It’s worth noting that researchers conducted a mendelian randomization study to further understand the pharmacological action of statins and discovered that rs12916 is closely associated with intricate metabolic changes related to statins, such as lipid reduction beyond LDL-C [33]. Interestingly, our findings showed that the rs12916 *CC* genotype is the independent risk factor of RCR (LDL-C > 1.8 mmol/L), which further supported the notion that variant rs12916 is connected to poor efficacy of statins and further enriched the understanding of this SNP rs12916. However, the detailed mechanisms are still not clear, which is worth further investigation in the future.

No association was observed between *NPC1L1* gene polymorphisms (rs11763759, rs4720470, rs2072183, and rs2073547) and RCR (LDL-C > 1.8 mmol/L) in the study. In spite of this, our previous study [11] showed that *NPC1L1* gene polymorphisms were associated with the prognosis of TVD. Inconsistent with our results, several previous studies [34–36] reported variant *NPC1L1* gene was associated with LDL-C reduction response to statins or a combination of statins and ezetimibe. The negative outcome in the present study may result from the populations treated with statins but without ezetimibe, since statin targets HMGCR while ezetimibe targets NPC1L1. Different study populations may also explain the discrepancy. In the future, we can further investigate whether these SNPs of the *NPC1L1* are associated with individual variations of the efficacy of ezetimibe in patients treated with ezetimibe.

In Chinese residents, the prevalence of dyslipidemia maintains at a high level, but the awareness rate, treatment rate and control rate of dyslipidemia are all at a low level. As for the RCR (LDL-C < 1.8 mmol/L), in other words, the standard-reaching rate of LDL-C, is also relatively low in the Chinese population. Consistent with the previous studies [37–39], the patients in our study reached the standard rate of LDL-C was also low, only 14.5% of the patients reached the LDL-C < 1.8 mmol/L, which caused the ratio of non-RCR group and RCR group nearly 1:6. To further reduce the risk of cardiovascular events in patients at high risk of CHD, the guidelines recommend that it is very important to reach the target of LDL-C as early as possible. Previously, there was no research to discuss the use of specific genetic testing to determine whether LDL-C achieves the target considerations after statins. Hence, the present study was the first to report that the risk of RCR increased to more than 2-fold in rs12916 genotype *CC* carriers, indicating that *HMGCR* genetic testing may be utilized to early screen patients with poor efficacy following moderate-intensity statins among those at high risk of CHD. For these people with poor efficacy, the early use of intensive statins or a combination of non-statin lipid-lowering drugs (e.g. ezetimibe or PCSK9i) can be considered in clinical practice to make LDL-C concentrations reach the target early and then reduce cardiovascular events risk, which may bring long-term benefits.

There was limited data on the genetic background of PTVD, especially the content of our two genes of interest, *HMGCR* and *NPC1L1*, and our study fills the gap in this field and might add something new to precision treatment of statins. There are some limitations to our study that should be noted. Firstly, all participants came from China. Whether the relationship exists among other ethnic groups needs to be further studied. Second, all participants have taken moderate-intensity statins, and the prescribed statins included atorvastatin, simvastatin, rosuvastatin, pivastatin, and fluvastatin. Although the target of all statins is HMGCR, whether the different sorts of statins may have an impact on the results needs to be further studied. Third, unmeasured confounding factors (such as the type of diet) still cannot be ruled out as related to the risk of RCR, although we attempted to adjust for as many important confounding factors as possible. Fourth, further bioinformatic analysis should be carried out in the future.

## Conclusion

To our knowledge, the present study first reported that carrying the homozygote (*CC*) for the minor allele of rs12916 within *HMGCR* gene was the independent risk factor of RCR (LDL-C > 1.8 mmol/L) in PTVD patients treated with moderate-intensity statins. Our results provided new insights into individualized lipid-lowering therapy. *HMGCR* genetic testing may be utilized to early screen patients with poor efficacy following moderate-intensity statins, and for these patients, initiating early use of intensive statins or a combination of non-statin lipid-lowering drugs may further reduce cardiovascular risk.

## Electronic supplementary material

Below is the link to the electronic supplementary material.

Additional File 1: Hardy-Weinberg equilibrium of genotype of *NPC1L1* and *HMGCR* genes

Additional File 2: Material

## Data Availability

The datasets generated and/or analysed during the current study are available in the ClinVar database of NCBI/NLM/NIH. The accession numbers for this submission are SCV002757991 - SCV002757997.

## References

[1] Report on Cardiovascular Health and Diseases in China. 2021: An Updated Summary. *Biomed Environ Sci* 2022, 35(7):573–603. 10.3967/bes2022.079 35945174

[2] Mihaylova B, Emberson J, Blackwell L, Keech A, Simes J, Barnes EH, Voysey M, Gray A, Collins R, Baigent C (2012). The effects of lowering LDL cholesterol with statin therapy in people at low risk of vascular disease: meta-analysis of individual data from 27 randomised trials. Lancet.

[3] Grundy SM, Stone NJ, Bailey AL, Beam C, Birtcher KK, Blumenthal RS, Braun LT, de Ferranti S, Faiella-Tommasino J, Forman DE (2019). 2018 AHA/ACC/AACVPR/AAPA/ABC/ACPM/ADA/AGS/APhA/ASPC/NLA/PCNA Guideline on the management of blood cholesterol: a report of the American College of Cardiology/American Heart Association Task Force on Clinical Practice Guidelines. Circulation.

[4] Giugliano RP, Cannon CP, Blazing MA, Nicolau JC, Corbalán R, Špinar J, Park JG, White JA, Bohula EA, Braunwald E (2018). Benefit of adding Ezetimibe to Statin Therapy on Cardiovascular Outcomes and Safety in patients with Versus without Diabetes Mellitus: results from IMPROVE-IT (improved reduction of outcomes: Vytorin Efficacy International Trial). Circulation.

[5] Sabatine MS, Giugliano RP, Keech AC, Honarpour N, Wiviott SD, Murphy SA, Kuder JF, Wang H, Liu T, Wasserman SM (2017). Evolocumab and Clinical Outcomes in patients with Cardiovascular Disease. N Engl J Med.

[6] Schwartz GG, Steg PG, Szarek M, Bhatt DL, Bittner VA, Diaz R, Edelberg JM, Goodman SG, Hanotin C, Harrington RA (2018). Alirocumab and Cardiovascular Outcomes after Acute Coronary Syndrome. N Engl J Med.

[7] Ridker PM (2017). How common is residual inflammatory risk?. Circ Res.

[8] Gao Y, Lou Y, Liu Y, Wu S, Xi Z, Wang X, Zhou Y, Liu W (2021). The relationship between residual cholesterol risk and plaque characteristics in patients with acute coronary syndrome: insights from an optical coherence tomography study. Atherosclerosis.

[9] Ference BA, Majeed F, Penumetcha R, Flack JM, Brook RD (2015). Effect of naturally random allocation to lower low-density lipoprotein cholesterol on the risk of coronary heart disease mediated by polymorphisms in NPC1L1, HMGCR, or both: a 2 × 2 factorial mendelian randomization study. J Am Coll Cardiol.

[10] Baigent C, Blackwell L, Emberson J, Holland LE, Reith C, Bhala N, Peto R, Barnes EH, Keech A, Simes J (2010). Efficacy and safety of more intensive lowering of LDL cholesterol: a meta-analysis of data from 170,000 participants in 26 randomised trials. Lancet.

[11] Zhao X, Li J, Tang X, Liu R, Xu J, Xu L, Jiang L, Huang K, Tian J, Feng X, et al. Association of NPC1L1 and HMGCR gene polymorphisms with major adverse Cardiac and cerebrovascular events in patients with three-vessel disease. Hum Gene Ther; 2021. 10.1089/hum.2020.229 33167740

[12] Collet JP, Zeitouni M, Procopi N, Hulot JS, Silvain J, Kerneis M, Thomas D, Lattuca B, Barthelemy O, Lavie-Badie Y (2019). Long-term evolution of premature coronary artery disease. J Am Coll Cardiol.

[13] Do R, Stitziel NO, Won HH, Jørgensen AB, Duga S, Angelica Merlini P, Kiezun A, Farrall M, Goel A, Zuk O (2015). Exome sequencing identifies rare LDLR and APOA5 alleles conferring risk for myocardial infarction. Nature.

[14] Jacobson TA, Ito MK, Maki KC, Orringer CE, Bays HE, Jones PH, McKenney JM, Grundy SM, Gill EA, Wild RA (2014). National lipid Association recommendations for patient-centered management of dyslipidemia: part 1 - executive summary. J Clin Lipidol.

[15] Miller SA, Dykes DD, Polesky HF (1988). A simple salting out procedure for extracting DNA from human nucleated cells. Nucleic Acids Res.

[16] Liu Y, Hu C, Liu C, Liu D, Mei L, He C, Jiang L, Wu H, Chen H, Feng Y (2019). A rapid improved multiplex ligation detection reaction method for the identification of gene mutations in hereditary hearing loss. PLoS ONE.

[17] Scholz M, Horn K, Pott J, Gross A, Kleber ME, Delgado GE, Mishra PP, Kirsten H, Gieger C, Müller-Nurasyid M (2022). Genome-wide meta-analysis of phytosterols reveals five novel loci and a detrimental effect on coronary atherosclerosis. Nat Commun.

[18] Zhao X, Xu J, Tang X, Huang K, Li J, Liu R, Jiang L, Zhang Y, Wang D, Sun K (2021). Effect of NPC1L1 and HMGCR genetic variants with premature triple-vessel coronary disease. Front Cardiovasc Med.

[19] Zhao X, Tang X, Xu J, Liu R, Huang K, Li J, Li Y, Jiang L, Xu L, Zhang Y (2022). Novel polymorphism of the HMGCR gene related to the risk of diabetes in premature triple-vessel disease patients. J Gene Med.

[20] Medina MW (2010). The relationship between HMGCR genetic variation, alternative splicing, and statin efficacy. Discov Med.

[21] Medina MW, Gao F, Ruan W, Rotter JI, Krauss RM (2008). Alternative splicing of 3-hydroxy-3-methylglutaryl coenzyme a reductase is associated with plasma low-density lipoprotein cholesterol response to simvastatin. Circulation.

[22] Chasman DI, Posada D, Subrahmanyan L, Cook NR, Stanton VP, Ridker PM (2004). Pharmacogenetic study of statin therapy and cholesterol reduction. JAMA.

[23] Krauss RM, Mangravite LM, Smith JD, Medina MW, Wang D, Guo X, Rieder MJ, Simon JA, Hulley SB, Waters D (2008). Variation in the 3-hydroxyl-3-methylglutaryl coenzyme a reductase gene is associated with racial differences in low-density lipoprotein cholesterol response to simvastatin treatment. Circulation.

[24] Polisecki E, Muallem H, Maeda N, Peter I, Robertson M, McMahon AD, Ford I, Packard C, Shepherd J, Jukema JW (2008). Genetic variation at the LDL receptor and HMG-CoA reductase gene loci, lipid levels, statin response, and cardiovascular disease incidence in PROSPER. Atherosclerosis.

[25] Donnelly LA, Doney AS, Dannfald J, Whitley AL, Lang CC, Morris AD, Donnan PT, Palmer CN (2008). A paucimorphic variant in the HMG-CoA reductase gene is associated with lipid-lowering response to statin treatment in diabetes: a GoDARTS study. Pharmacogenet Genomics.

[26] Singer JB, Holdaas H, Jardine AG, Fellstrøm B, Os I, Bermann G, Meyer JM (2007). Genetic analysis of fluvastatin response and dyslipidemia in renal transplant recipients. J Lipid Res.

[27] Chien KL, Wang KC, Chen YC, Chao CL, Hsu HC, Chen MF, Chen WJ (2010). Common sequence variants in pharmacodynamic and pharmacokinetic pathway-related genes conferring LDL cholesterol response to statins. Pharmacogenomics.

[28] Swerdlow DI, Preiss D, Kuchenbaecker KB, Holmes MV, Engmann JE, Shah T, Sofat R, Stender S, Johnson PC, Scott RA (2015). HMG-coenzyme A reductase inhibition, type 2 diabetes, and bodyweight: evidence from genetic analysis and randomised trials. Lancet.

[29] Schooling CM, Au Yeung SL, Zhao JV (2022). Exploring Pleiotropic Effects of lipid modifiers and targets on measures of the Coagulation System with Genetics. Thromb Haemost.

[30] Sarsenbayeva A, Jui BN, Fanni G, Barbosa P, Ahmed F, Kristófi R, Cen J, Chowdhury A, Skrtic S, Bergsten P et al. Impaired HMG-CoA reductase activity caused by genetic variants or statin exposure: impact on human adipose tissue, β-Cells and metabolome. Metabolites 2021, 11(9). 10.3390/metabo11090574 PMC8468287 34564389

[31] Schroor MM, Mokhtar FBA, Plat J, Mensink RP. Associations between SNPs in intestinal cholesterol absorption and endogenous cholesterol synthesis genes with cholesterol metabolism. Biomedicines 2021, 9(10). 10.3390/biomedicines9101475 PMC8533139 34680591

[32] Kettunen J, Holmes MV, Allara E, Anufrieva O, Ohukainen P, Oliver-Williams C, Wang Q, Tillin T, Hughes AD, Kähönen M (2019). Lipoprotein signatures of cholesteryl ester transfer protein and HMG-CoA reductase inhibition. PLoS Biol.

[33] Würtz P, Wang Q, Soininen P, Kangas AJ, Fatemifar G, Tynkkynen T, Tiainen M, Perola M, Tillin T, Hughes AD (2016). Metabolomic profiling of statin use and genetic inhibition of HMG-CoA reductase. J Am Coll Cardiol.

[34] Simon JS, Karnoub MC, Devlin DJ, Arreaza MG, Qiu P, Monks SA, Severino ME, Deutsch P, Palmisano J, Sachs AB (2005). Sequence variation in NPC1L1 and association with improved LDL-cholesterol lowering in response to ezetimibe treatment. Genomics.

[35] Hegele RA, Guy J, Ban MR, Wang J (2005). NPC1L1 haplotype is associated with inter-individual variation in plasma low-density lipoprotein response to ezetimibe. Lipids Health Dis.

[36] Polisecki E, Peter I, Simon JS, Hegele RA, Robertson M, Ford I, Shepherd J, Packard C, Jukema JW, de Craen AJ (2010). Genetic variation at the NPC1L1 gene locus, plasma lipoproteins, and heart disease risk in the elderly. J Lipid Res.

[37] Zhang M, Deng Q, Wang L, Huang Z, Zhou M, Li Y, Zhao Z, Zhang Y, Wang L (2018). Prevalence of dyslipidemia and achievement of low-density lipoprotein cholesterol targets in chinese adults: a nationally representative survey of 163,641 adults. Int J Cardiol.

[38] Zeng YY, Liu J, Liu J, Hao YC, Yang N, Zhou MG, Hu GL, Zhao D. [The expanding needs on lipid-lowering treatment in patients with acute coronary syndrome by applying newly issued definition of extreme high-risk by Chinese Society of Cardiology]. In: *Zhonghua Xin Xue Guan Bing Za Zhi* vol. 48, 2020/12/29 edn; 2020: 1039–1046. 10.3760/cma.j.cn112148-20200710-00549 33355748

[39] Li S, Liu HH, Guo YL, Zhu CG, Wu NQ, Xu RX, Dong Q, Li JJ (2021). Improvement of evaluation in chinese patients with atherosclerotic cardiovascular disease using the very-high-risk refinement: a population-based study. Lancet Reg Health West Pac.

