# Supplementary material for: HMGCR gene polymorphism is associated with residual cholesterol risk in premature triple-vessel disease patients treated with moderate-intensity statins
Source: BMC Cardiovasc Disord. 2023 Jun 24;23:317. doi: 10.1186/s12872-023-03285-w (PMC10290797; doi:10.1186/s12872-023-03285-w)
Supplement: Supplementary file 1 — Additional File 1: Hardy-Weinberg equilibrium of genotype of NPC1L1 and HMGCR genes [file 12872_2023_3285_MOESM1_ESM.docx]

**Table S1.** Hardy–Weinberg equilibrium of genotype of *NPC1L1* and *HMGCR* genes

| **Gene** | **SNPs** | **Major allele** | **Minor allele** | **Frequency of Genotype** | **Homozygote for the major allele (%)** | **Heterozygote (%)** | **Homozygote for the minor allele** | **HWE-*P* value** |
| --- | --- | --- | --- | --- | --- | --- | --- | --- |
| *NPC1L1* | rs11763759 | *T* | *C* |  | *TT* | *CT* | *CC* |  |
|  |  | 1155/1218 (0.948) | 63/1218 (0.052) | Observed value | 549 (90.1) | 57 (9.4) | 3 (0.5) | 1.000* |
|  |  |  |  | Expected value | 547 (89.9) | 60 (9.9) | 2 (0.3) |  |
|  | rs4720470 | *C* | *T* |  | *CC* | *CT* | *TT* |  |
|  |  | 826/1218 (0.678) | 392/1218 (0.322) | Observed value | 286 (47.0) | 254 (41.7) | 69 (11.3) | 0.734 |
|  |  |  |  | Expected value | 280 (46.0) | 266 (43.7) | 63 (10.4) |  |
|  | rs2072183 | *G* | *C* |  | *GG* | *CG* | *CC* |  |
|  |  | 773/1218 (0.635) | 445/1218 (0.365) | Observed value | 242 (39.7) | 289 (47.5) | 78 (12.8) | 0.923 |
|  |  |  |  | Expected value | 246 (40.3) | 282 (46.7) | 81 (13.3) |  |
|  | rs2073547 | *A* | *G* |  | *AA* | *GA* | *GG* |  |
|  |  | 771/1218 (0.633) | 447/1218 (0.367) | Observed value | 241 (39.6) | 289 (47.5) | 79 (13.0) | 0.936 |
|  |  |  |  | Expected value | 244 (40.1) | 283 (46.5) | 82 (13.5) |  |
| *HMGCR* | rs12916 | *T* | *C* |  | *TT* | *CT* | *CC* |  |
|  |  | 594/1218 (0.488) | 624/1218 (0.512) | Observed value | 157 (25.8) | 280 (46.0) | 172 (28.2) | 0.387 |
|  |  |  |  | Expected value | 145 (23.8) | 304 (50.0) | 160 (26.2) |  |
|  | rs2303151 | *C* | *T* |  | *CC* | *CT* | *TT* |  |
|  |  | 984/1218 (0.808) | 234/1218 (0.192) | Observed value | 396 (65.0) | 192 (31.5) | 21 (3.4) | 0.976 |
|  |  |  |  | Expected value | 398 (65.3) | 189 (31.0) | 22 (3.7) |  |
|  | rs4629571 | *A* | *G* |  | *AA* | *GA* | *GG* |  |
|  |  | 1103/1218 (0.906) | 115/1218 (0.094) | Observed value | 500 (82.1) | 103 (16.9) | 6 (1.0) | 1.000 |
|  |  |  |  | Expected value | 500 (82.1) | 104 (17.0) | 5 (0.9) |  |

***Note:*** *HMGCR, 3-hydroxy-3-methylglutaryl-coenzyme A reductase; NPC1L1, Niemann-Pick C1-like 1;*

*Compared by Fisher’s exact test.
