# Supplementary material for: HMGCR gene polymorphism is associated with residual cholesterol risk in premature triple-vessel disease patients treated with moderate-intensity statins
Source: BMC Cardiovasc Disord. 2023 Jun 24;23:317. doi: 10.1186/s12872-023-03285-w (PMC10290797; doi:10.1186/s12872-023-03285-w)
Supplement: Supplementary file 2 — Additional File 2: Material [file 12872_2023_3285_MOESM2_ESM.docx]

**Supplementary method**

Briefly, 100–200 ng of DNA sample was first denatured at 98 ℃ for 5 min in a 10 μL reaction containing 2.5 μL 4x DNA lysis buffer and then mixed well with a 10 μL ligation premix composed of 2 μL 10x ligase buffer, 0.5 μL ligase, 1 mL probe mix, and 7.5 μL Milli-Q water. The ligation reaction was carried out in an ABI2720 thermal cycler. The ligation cycling program was 4 cycles x (94℃ 1 min, 58℃ 4 h); 94℃ 2 min; hold at 72℃. Two 48-plex fluorescence PCR reactions were performed for each ligation product. PCR reactions were prepared in a 20 μL mixture containing 2x PCR master mix, 1 μL primer mix set A or set B, and 1 μL ligation product. The PCR cycling conditions were as follows: 95℃ for 2 min; followed by 9 cycles of 94℃ for 20 s, 65-0.5℃/cycle for 40 s, and 72℃ 1 min 30 s; then 25 cycles of 94℃ for 20 s, 57℃ for 40s, and 72℃ 1 min 30 s; and a final extension of 68℃ for 60 min and holding at 4℃. PCR products were separated and detected by capillary electrophoresis in an ABI3730XL sequencer. Raw data were analyzed according to the information obtained for the labeling dye color and fragment size of the allele-specific ligation-PCR product. Genotyping was conducted without any knowledge regarding the subject’s case or control status. For quality control, repeated analyses were accomplished to guarantee the genotyping quality by randomly choosing 4 % of samples with high DNA quality.
